# Learning walks in an Australian desert ant, *Melophorus bagoti*

**DOI:** 10.1242/jeb.242177

**Published:** 2021-08-26

**Authors:** Sudhakar Deeti, Ken Cheng

**Affiliations:** Department of Biological Sciences, Macquarie University, Sydney, NSW 2109, Australia

**Keywords:** Forager, Landmark learning, Visual navigation, Pirouettes, Voltes, Scans

## Abstract

The central Australian ant *Melophorus bagoti* is the most thermophilic ant in Australia and forages solitarily in the summer months during the hottest period of the day. For successful navigation, desert ants of many species are known to integrate a path and learn landmark cues around the nest. Ants perform a series of exploratory walks around the nest before their first foraging trip, during which they are presumed to learn about their landmark panorama. Here, we studied 15 naive *M. bagoti* ants transitioning from indoor work to foraging outside the nest. In 3–4 consecutive days, they performed 3–7 exploratory walks before heading off to forage. Naive ants increased the area of exploration around the nest and the duration of trips over successive learning walks. In their first foraging walk, the majority of the ants followed a direction explored on their last learning walk. During learning walks, the ants stopped and performed stereotypical orientation behaviours called pirouettes. They performed complete body rotations with stopping phases as well as small circular walks without stops known as voltes. After just one learning walk, these desert ants could head in the home direction from locations 2 m from the nest, although not from locations 4 m from the nest. These results suggest gradual learning of the visual landmark panorama around the foragers’ nest. Our observations show that *M. bagoti* exhibit similar characteristics in their learning walks to other desert ants of the genera *Ocymyrmex* and *Cataglyphis*.

## Abstract (precis)

**Summary:** Before becoming foragers, *Melophorus bagoti* ants took 3–7 learning walks around their nest. They increased the duration and area explored over successive walks, stopping occasionally to scan the environment.

## INTRODUCTION

Desert ants lead a thermophilic life on multiple continents and are excellent experimental models for insect navigation (Wehner, 1987, 2020). Desert ants are found in Southern Africa (*Ocymyrmex*), North Africa and Eurasia (*Cataglyphis*), South America (*Dorymyrmex*) and Australia (*Melophorus*; Cheng et al., 2014; Wehner, 1987, 2003; Wehner et al., 1992). Like other thermophilic desert ants, *Melophorus bagoti* foragers have short lives outside the nest in which they accomplish remarkable visual navigational tasks. They possess a navigational toolbox containing multiple strategies, relying on the acquisition of multiple cue sources (Freas et al., 2019). The most well-studied components of the toolbox are path integration via a celestial compass (Collett and Collett, 2000; Wehner, 2008), landmark guidance based primarily on visual cues between their nest and a profitable foraging area (Cheng et al., 2009; Collett et al., 2006; Wehner, 2008), and systematic searching (Schultheiss and Cheng, 2011; Schultheiss et al., 2013; Wehner and Srinivasan, 1981; Wystrach et al., 2013).

Recently, learning walks around the nest of inexperienced foragers have received more attention (Collett and Zeil, 2018; Zeil and Fleischmann, 2019). During the transition from indoor to outdoor life and before becoming foragers, ants learn the surrounding panorama around the nest through several pre-foraging learning walks by meandering in small loops around the nest entrance (Fleischmann et al., 2016; Jayatilaka et al., 2018; Müller and Wehner, 2010; Muser et al., 2005; Wehner et al., 2004). Small exploratory walks are a prerequisite for successful homing (*Cataglyphis fortis*: Fleischmann et al., 2016; *Myrmecia croslandi*: Jayatilaka et al., 2018; for review, see Freas et al., 2019). With increasing experience, the duration of the ants’ trips increases and the trips cover a greater area than previous trips (Fleischmann et al., 2016). During learning walks, the ants stop and perform stereotypical orientation behaviours towards the nest or other places (Fleischmann et al., 2016; Freas et al., 2019; Jayatilaka et al., 2018; Müller and Wehner, 2010). Studies on *Cataglyphis noda* and *Cataglyphis aenescens* showed that they perform body rotations with stopping phases, called pirouettes, with the longest stopping phases facing the direction of the nest, as well as small circular walks without stops known as voltes (*C. fortis*) (Fleischmann et al., 2017). The naive ants perform a complete (±360 deg) or partial (±180 deg) turn about their body axis in a pirouette. The pirouettes are often interrupted by distinct stopping phases during which the ant does not move forward and keeps its gaze within a range of 10 deg for at least 100 ms. In a pirouette, the learner performs a complete or partial turn around the body axis while stopping periodically facing different directions. This is most likely to learn views of the distant panorama around the nest entrance. With the voltes, the ant walks a small circle in a rotational movement without stopping. This movement may enable celestial cues to be calibrated for navigational purposes. However, the role of these turns in navigation remains unclear.

Ants do learn the nest panorama (Narendra et al., 2007). Learning the panorama of the nest is critical for *M. bagoti* foragers because they are known to forage long distances from the nest site (Muser et al., 2005; Schultheiss and Nooten, 2013). They also need to learn several panoramas along the foraging route and at profitable food sites for successful navigation (Freas et al., 2017; Graham and Cheng, 2009). It is assumed that the foragers learn when they look back occasionally on their first foraging trips away from the nest (Freas and Cheng, 2018; Zeil et al., 2014). When leaving a profitable food source on the first trip, *M. bagoti* foragers showed turn-back-looks towards the feeder (Freas and Cheng, 2018). In addition, desert ants showed that they were able to home from other non-visited locations up to 10 m away after learning the nest panorama in a limited area around their nest entrance (Deeti et al., 2020; Fleischmann et al., 2018a; Wystrach et al., 2012). Earlier studies on *M. bagoti* also noted their exploration walks during the first 2 days of outdoor life (Muser et al., 2005), finding that they turn and stop in a pirouette-like fashion as well. These studies on *M. bagoti* ants consistently show the importance of visual landmarks in navigation and nest finding. Further research is needed, however, to understand the ontogenetic nature of learning walks of these desert ants in detail.

In the present research, we detailed the learning walks in the Australian desert ant, *Melophorus bagoti*, in their natural terrain. The structure and spatial distribution of learning walks at the nest site were recorded. The scans found in learning walks were studied with the aim of understanding how space is organised. We followed the performance of individually identified *M. bagoti* ants’ learning walks from their first appearance until the ants went off to forage. We noted the scanning patterns of the ants during their learning walks. Additionally, we tested a separate group of ants on their ability to use the terrestrial panorama after their first learning walk.

## MATERIALS AND METHODS

### Test animals and study site

**Fig. 1.**
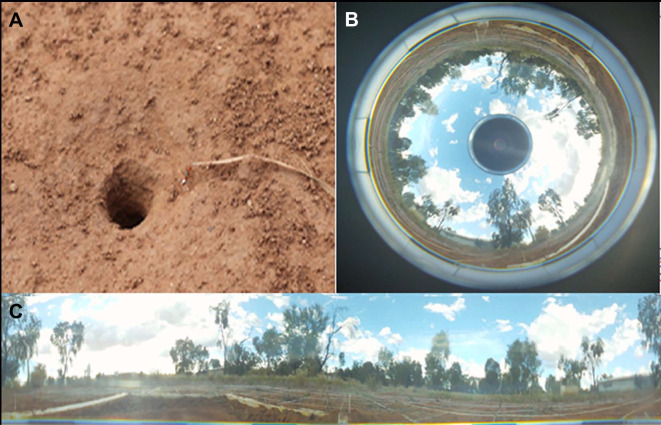
**Nest and terrestrial landmarks.** (A) Entrance of the *Melophorus bagoti* nest used in the experiment, showing one painted ant. (B,C) Panoramic 360 deg image at the nest of the surrounding view. Panoramic images were taken with a Sony HD Bloggie camera (B) and converted into a cylindrical image using My Home Memories software (C). The area immediately around the nest was open, allowing distant vistas of trees and buildings.

In the summer of November 2018 to February 2019, we performed a study on *Melophorus bagoti* Lubbock 1883 desert ants from a single nest in the grounds of the Centre for Appropriate Technology, which is 10 km south of Alice Springs, NT, Australia (23°45′28.12″S, 133°52′59.77″E). The vegetation of this semi-arid desert habitat is dominated by buffel grass (*Pennisetum cenchroides*), a mosaic of Acacia bushes, and Eucalyptus trees (Fig. 1). No Australian ethical regulations are stipulated for studying ants and the experimental procedures were completely non-invasive. This study required naive ants. The average foraging span of *M. bagoti* outside the nest is 4.9 days (Muser et al., 2005). Therefore, it was assumed that ants appearing after 5 days had passed were naive. To identify these naive ants, for a period of 5 days all foragers of the colony were painted with the same colour (Deeti and Cheng, 2021). All the unpainted ants of the nest appearing on the 6th day and thereafter were considered as newcomers that had not had any experience with terrestrial landmarks around their nest. These individuals were caught at the nest entrance and marked with a unique pattern of paints (Tamiya™) on their body (Deeti and Cheng, 2021). After painting the naive ants, we released them into the nest entrance; we blocked the nest entrance by placing a lid on it to effectively prevent the ant from coming back outside (Deeti and Cheng, 2021).

### Experimental procedure

The vegetation around the selected nest was removed on the first day of the study. On the 6th morning, a 10×10 m grid was established around the nest entrance by using synthetic white string that was wound around pegs in the ground. This was necessary for plotting the paths of the naive ants. A 2×2 m area around the nest was subdivided into 0.25 m squares and beyond this area 1 m squares were gridded. As the ant moved around the white string grid on the ground, its path was drawn on a similar grid on a piece of graph paper. To characterise the learning walks of *M. bagoti*, on leaving the nest for the first time, i.e. the first run after being individually painted, learning walks of individual ants (*n*=15) were recorded on the gridded paper. Each and every walk of the focal ants was recorded until they crossed the 10×10 m area and went off to forage. When an ant travelled beyond the 10×10 m area, we considered it as a forager as all such ants went much farther from the nest. These first foraging walks concluded an ant's participation in the study and its subsequent paths were not recorded. We mostly observed one ant at one time. On only one occasion did two ants appear at the same time. One ant was followed and the other ant went off to forage. We estimated this other ant's foraging direction. We recorded the individual ants’ time and day of appearance. For each learning walk, we also recorded the locations where pirouettes were performed. In addition, the panoramic view around the nest was captured at the nest entrance. The ants foraged during the day time, the activity ceasing during the twilight evening, entire night and early morning. Each evening, the ants closed their nest entrance. We covered the nest entrance overnight in order to record all appearances of the ants, i.e. all learning walks outside the nest on the morning of the next day.

In addition, by using a video camera (EOS 80D Canon), we captured the learning walks of a few painted ants. The video camera was mounted on a tripod and placed on the north side of the nest entrance. Because of high temperatures in Alice Springs, the camera stopped working after ∼20 min and failed to record some of the ant activity. After the camera stopped for a while, it would function normally again and thus once again recorded ant activity. The tripod was left at the same location to keep the landmarks similar for all ants. The full HD video camera recorded an area around the nest of about 50×40 cm at 50 frames s^−1^ with an image size of 1920×1280 pixels. This video camera recording was turned on remotely via live-view shooting (Canon Camera Connect 2.3.20 version) whenever focal ants appeared. We managed to record eight first learning walks and six second learning walks of the ants.

The displacement experiment was conducted on 56 naive ants from the same nest. These tested ants were a different cohort from the ants observed for learning walks. After their first learning walk, these new (unpainted) naive ants were caught before entering the nest and displaced to two test locations at 2 or 4 m displacement sites that were south and north of the nest. After testing, ants were painted with black colour. Each ant was tested at the same distance from the nest entrance, once to the north and once to the south of the nest, in random order. The 2 m displacement tests were done on one group of ants (*n*=29) followed by 4 m displacement tests on a different group of ants (*n*=27). To quantify the direction of initial orientations at displacement sites, ants were released on a goniometer. The goniometer contained a 60 cm-diameter circle drawn on a wooden board and separated into 24 equal 15 deg wedges. The initial orientations were determined by measuring the wedge at which ants first crossed the circumference 30 cm from the centre (Deeti et al., 2020; Freas and Cheng, 2017). Test ants were captured manually near their nest with the help of an Eppendorf tube, transported in darkness, and released on the centre of the goniometer. During the tests, ants were released one at a time on the goniometer. After leaving the goniometer of the first test, the ants were captured in a test tube and released at the second site.

### Data analysis

The learning walks and body saccades were easily identifiable with the naked eye and therefore their locations were noted on paper. The recorded learning walks of focal ants on grid paper sheets were scanned at a resolution of 300 dpi for digitisation. The resulting images were then digitised in GraphClick (www.arizona-software.ch/graphclick) to convert scanned paths into *x*–*y* coordinates. The nest location was chosen as the origin (0, 0). From the digitised data, the area of exploration of the ant was calculated in a customised R programming function. The area of exploration of the walk is defined as the space inside the path travelled by the ant from the first point to the last point with reference to the nest. The maximum distance from the nest travelled by the ant was calculated. We also calculated the number of head rotations between 1 and 360 deg during the trips outside the nest, based on data recorded on the paper. Each individual learning-walk duration was also noted.

From the video tapes, we calculated the time interval between scanning bouts of eight first learning walks and of six second learning walks. In subsequent walks, ants moved out of the recording area and we did not analyse those data. A scanning bout was defined as a stop with saccadic body rotations (scans) performed at one spot. We also calculated the durations of stopping phases of ants and the durations of tight circular turns without stopping phases (voltes) from the video tapes. As we could not place the camera in a perpendicular position over the nest, however, we could not analyse the orientation directions of the scans or gauge angular velocities accurately.

To show the path parameters more clearly, we transformed the data of area, maximum distance, duration and number of pirouettes to a log scale. The differences in the path parameters across walks were analysed using a one-way repeated-measures ANOVA across walks 1–4 (*n*=12). Including any more walk numbers would lead to too low a sample size for statistical analysis, because a repeated-measures ANOVA would have to include only ants that had completed five or more walks. Then, we used linear or quadratic regression models to fit the data and estimate the association between the learning walk number and path parameters (dependent variables: path parameters; independent variable: walks; random factor: individual ants). Durations of turning and stopping phases during scans in partial versus complete body rotations were analysed using Welch's paired *t*-test. In order to understand the relationship between the pivoting direction (clockwise or anticlockwise) and path curvature (curving to the left or right), we performed the Chi-squared test (dependent variable: pivoting direction, clockwise or anticlockwise; independent variable: curvature of the path before or after the pivots, to the left or to the right). The circular correlation between the centroid directions of the last learning walk and first foraging walk was measured and calculated according to Fisher and Lee's correlation formula (Fisher and Lee, 1983).

Test ants’ initial orientations on displacement tests were analysed with circular statistics using the statistical program Oriana Version 4 (Kovach Computing Services™). To test for a uniform distribution of headings (*P*>0.05) for each condition, a Rayleigh's test was conducted. To test whether initial orientations were significantly clustered around the nest direction at 0 deg, we examined whether 0 deg fits within the 95% confidence interval (CI) of orientations (Watson tests) and conducted *V*-tests, with alpha set at *P*=0.05. A *V*-test delivers a significant result when a distribution of mean headings is notably clustered around a specified target direction. We also conducted single-sample log likelihood ratio tests to examine whether the heading distributions of the ants are uniform in each test condition.

## RESULTS

We chose 15 naive ants from a single ant colony to examine the learning walks of the Australian desert ant *M. bagoti* before they went foraging. We observed three kinds of movements near the nest, other than foragers heading off. Firstly, ants excavated their nest: they disposed of sand granules within 5–10 cm of the nest entrance. Secondly, ants disposed of waste materials such as foraged food: they carried the unwanted materials outside the nest, travelled in a straight path, and then dropped the materials and returned straight to the nest. The foragers, non-foragers and guard ants participated in these activities at the start of the day. Thirdly, ants performed learning walks, detailed below. They started with a tiny path length forming loops near the nest combined with turn-back looks by which the learners repeatedly faced the general direction of the nest entrance. The turns were either full (360 deg) rotations about the ant's vertical body axis or partial (<360 deg) rotations.

**Fig. 2.**
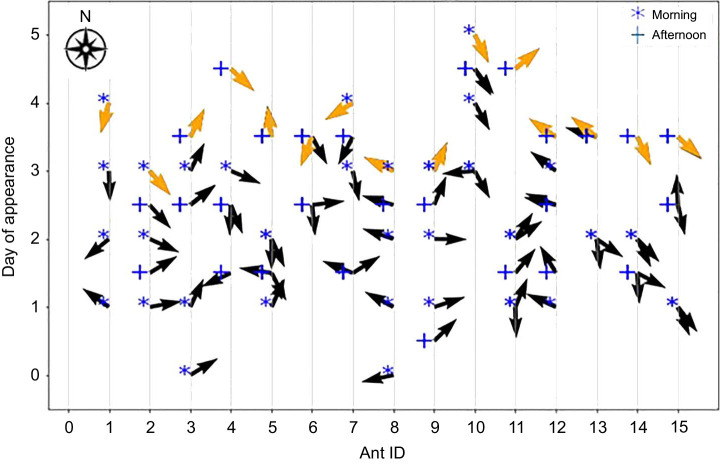
**Distribution of learning walks over time.** The time of learning walks of all 15 ants and the centroid direction of each walk. The tick marks on the *y*-axis show the time of appearance of the ants after being painted (morning and afternoon as indicated); 0 corresponds to 09:00 h of the day that the first focal ant appeared. The arrows indicate the centroid direction of each learning walk, defined as the direction of the average of all the points (centroid) on the walk. The top arrow in gold represents an ant's first foraging trip. Arrows close to one another indicate an ant re-appearing after a short delay.

After naive ants were painted and released in the nest, three out of 15 ants reappeared on the same day and performed their first learning walk (day 0 in Fig. 2), whereas nine ants reappeared on the next day (day 1), two ants after 2 days (on day 2) and one ant after 3 days (on day 3). The number of walks taken by the ants before their first foraging trip ranged from 3 to 7 (Fig. 2). For six ants, the time of their first learning walk and the time of their first foraging walk were in different periods of the day (morning or afternoon) and nine ants conducted their first learning walk and first foraging walk both in the morning or in the afternoon (Fig. 2). The ants performed most of the walks 2 and 3 days after their first learning walk. Only three ants took 4 days before starting to forage (Fig. 2).

**Fig. 3.**
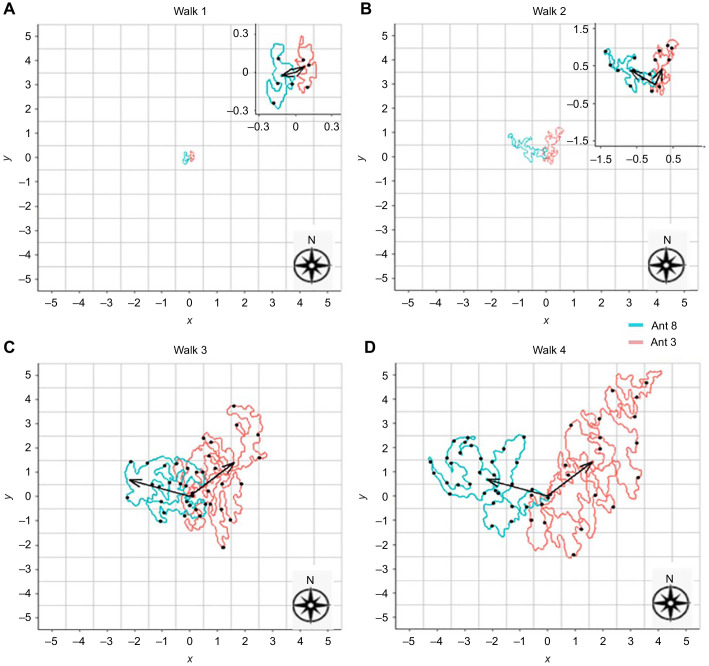
**Representative examples of successive learning walks of *M. bagoti*.** (A–D) Paths from walk 1 to walk 4 of two individual ants, shown on a 10×10 m grid. (0, 0) is the nest position. For clarity, ants with walks concentrated in different directions were chosen. Each unit on the axes is 1 m. The length of each arrow denotes the distance of the centroid from the origin (nest position) whereas the angle of each arrow shows the centroid direction of the path, with short arrows shown in the insets of walk 1 and walk 2. Black dots indicate the positions of scanning bouts during the learning walks.

**Fig. 4.**
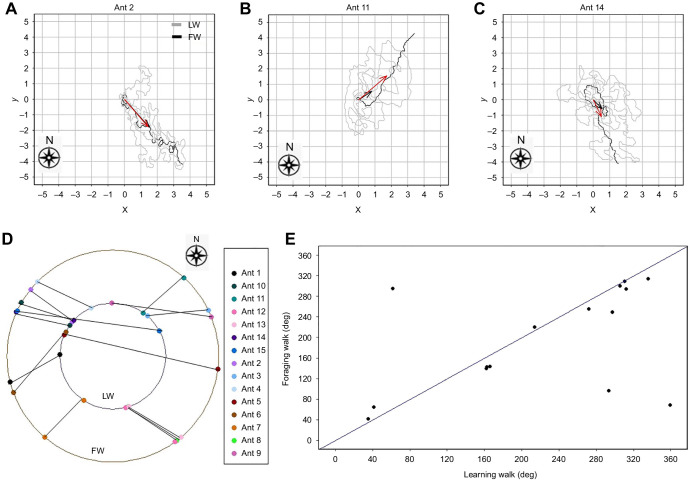
**Correlation between the last learning walks and first foraging walks.** (A–C) The last learning walks (LW) and first foraging walks (FW) of three focal ants, shown on 10×10 m grids. (0, 0) is the nest position. Each unit on the axes is 1 m. The black and red arrows indicate the centroid directions of the last learning walk and first foraging walk, respectively. (D,E) The circular correlogram of the last learning walk and first foraging walk of each ant, represented in two ways. D shows walk directions arrayed on concentric circles with corresponding pairs from the same ant identified by colour and connected by a line segment. E shows a toroid (donut) shape that has been unwrapped to be displayed on a plane. Thus, the right edge (360 deg) wraps around to the left edge (0 deg) and the top edge (360 deg) wraps around to the bottom edge (0 deg). It can be seen that 12 of 15 points (ants) fall near the diagonal line, which indicates the line of equal (same) centroid directions on the two walks.

For each learning walk of each individual ant, the average *x* and *y* coordinates from the start of the journey until the ant returned to the nest were calculated. These averages defined the path centroid whose direction is shown in Fig. 2. Out of 15 ants, 12 started their first foraging trip in the sector of the centroid of their last learning walk (±45 deg of the centroid direction, Fig. 2; *P*<0.001 by a binomial test). Some ants concentrated their learning walks in a particular sector (for example, ant 3 in Fig. 3). Altogether, five ants followed the sector containing the centroid direction of their first learning walk in their all subsequent learning walks and chose a direction in the same sector on their first foraging walks. Eight ants performed at least two exploratory walks in the ±45 deg sector of the direction of their first foraging walk (Figs S1 and S2). The circular correlation (*R*^2^=0.79) between the directions of last learning walks and first foraging trips across all ants is shown on two concentric circles in Fig. 4D and on a plane representing an unwrapped torus in Fig. 4E, along with representative examples (Fig. 4A–C) and data for all 15 ants (Fig. S2). The unwrapped torus (Fig. 4E) shows clearly that 12 of 15 ants fall near the line of equality for last foraging walk direction and first learning walk direction (diagonal line).

**Fig. 5.**
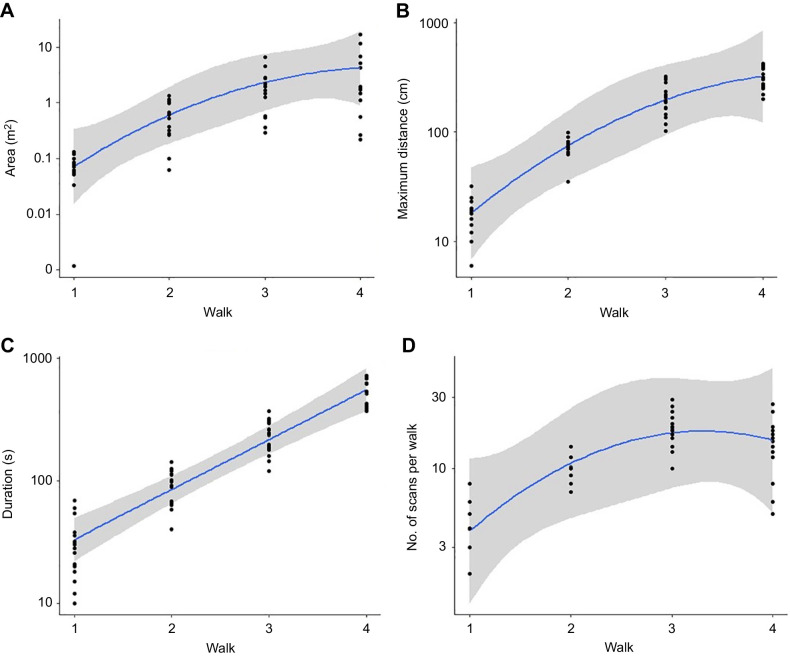
**Learning walk characteristics.** The ants’ area of exploration (A), maximum distance from the nest (B), walk duration (C) and number of scans on learning walks (each pause after a turn on the spot is counted as a scan, D) in their first four learning walks (*y*-axis values are plotted on a log_10_ scale). Quadratic best-fit lines have been added with 95% confidence intervals in grey.

In the first learning walk, ants travelled near the nest, returning by walking in a circle close to the nest (Fig. 3A; Fig. S1A). The total area around the nest covered by the ants from the start of the trip to the end of the trip was 0.07±0.035 m^2^ (mean±s.d.). The distance between the farthest point and the nest entrance measured 18.4±6.36 cm. The ants travelled for 30.86±17.86 s before completing their first walks. In the first learning walk, an ant's walking area did not cover all the compass directions before it returned to the nest (Fig. 3A; Fig. S1A). Combining all ants across all their learning walks, the ants walked the most near their nest, progressively decreasing their coverage with distance from the nest (Fig. S3). From the first walk to the second walk, the maximum distance travelled increased on average 3.8-fold, and in each consecutive walk the maximum distance gradually increased up to ∼2-fold (Fig. 5B). The area covered by ants around the nest increased 8-fold in their second walk, and the ants systematically covered all the areas around the nest. Furthermore, in the subsequent walks, the area rose steadily up to 2.5-fold. (Fig. 5A). The duration of learning walks also increased ∼4-fold in the course of their second learning walk and likewise it increased ∼2-fold in the successive walks until the ants went foraging (Fig. 5C). In inferential statistics, the maximum distance from the nest increased significantly from walk 1 to walk 4 (one-way ANOVA *F*_3,44_=88.5, *P*<0.0005, linear trend: *F*_1,44_=88.5, *P*<0.0005, quadratic trend: *F*_1,44_=38, *P*<0.0005). With successive walks, the ants covered a larger area (one-way ANOVA *F*_3,44_=7.17, *P*<0.0005, linear trend: *F*_1,44_=7.17, *P*<0.0005, quadratic trend: *F*_1,44_=3.8, *P*<0.01) and walked for longer (one-way ANOVA *F*_3,44_=87.9, *P*<0.0005; linear trend: *F*_1,44_=87.9, *P*<0.0005; quadratic trend: *F*_1,44_=33.7, *P*=0.0153).

During their learning walks, *M. bagoti* showed two different types of scans. Ants stopped and performed saccadic body and head movements, pivoting systematically towards the nest or surrounding landmarks in scans or pirouettes. The complete sequence of stopping at one spot and pivoting several times is called a scanning bout. In the process, the ants performed a series of complete or partial rotations with their body to look back at the nest entrance or look at the surrounding panorama. Ants also walked around in small tight circles without stopping, called voltes. The number of scans per learning walk was quantified for walks 1–4 (Fig. 5D). We also plotted the positions of scanning bouts across the learning walks (Fig. 3; Fig. S4A,B). From walk 1 to walk 2, the number of scans increased 2.6-fold and scans taken by the ants increased until walk 3 and then decreased. There was a significant difference across successive learning walks in the number of scans (*F*_3,44_=21.51, *P*<0.0005, linear trend: *F*_1,44_=47.23, *P*<0.0005, quadratic trend: *F*_1,44_=21.51, *P*<0.0001). During the learning walks, *M. bagoti* performed more pirouettes than voltes, with only seven voltes observed from 15 ants across all learning walks. In the first foraging trip, nine ants stopped and scanned before setting off. The number of stop and scans, however, was fewer than in learning walks.

**Fig. 6.**
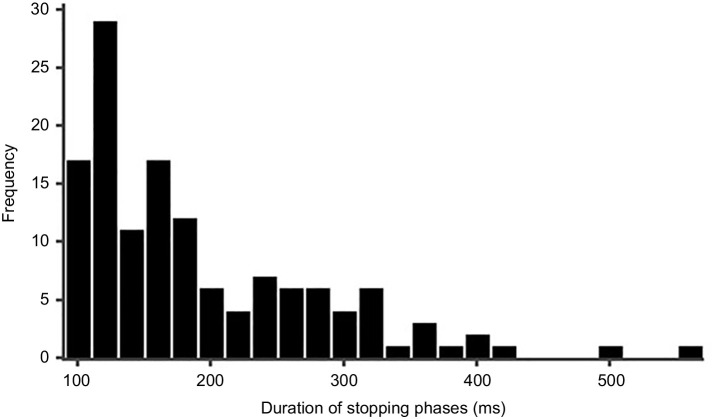
**Stopping phases of scanning bouts.** The frequency distribution of durations of stopping phases in scanning bouts during the first two learning walks. Data were put into bins of 20 ms (dictated by the frame rate of the camera), starting at the minimum stopping duration of 90–110 ms.

**Fig. 7.**
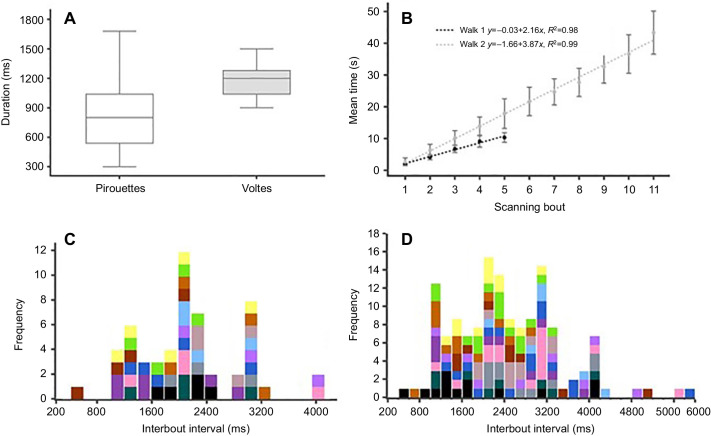
**Timing of scanning bouts.** (A) Durations of entire bouts of pirouettes and voltes across learning walks captured on video. The box plots show the median (middle line in the box), the lower and upper quartiles of the box, and observations outside the interquartile range (whiskers). (B) The mean (±s.d.) time of scanning bouts, measured from the start of the video recording, within 1 s of the appearance of the focal ant (0 on the *y*-axis), from walk 1 and walk 2. The dotted lines (black and grey) show the best linear fits. (C,D) The individual distributions of interbout intervals of scanning bouts in walk 1 (C) and walk 2 (D). Each colour represents one individual ant.

From the video recordings, we quantified the duration of stopping phases and rotation phases in both complete and partial pirouettes (Fig. S4C) and the frequency of left and right turns during scans in the first two learning walks (Fig. S4D). Paired-samples *t*-tests of mean duration of stopping phases of complete and partial pirouettes (*t*_13_=−1.51, *P*=0.55) and rotation phases (*t*_13_=−0.95, *P*=0.34) showed no significant differences in the durations of these phases in pirouettes from complete and partial rotations. Ants made similar numbers of left and right turns in total (Fig. S4D). Within a single scanning bout, however, ants always turned in one direction from saccade to saccade. At the suggestion of a reviewer, we examined the proportion of right-turning and left-turning scanning bouts as a function of whether the path preceding and following the bout curved to the left or right (Fig. S5). Proportions of right-turning and left-turning saccades were similar following left- and right-curving paths, but ants tended predominantly to continue to turn their paths by curving in the same direction as the turning direction of saccades. With a small number of ants and a small number of bouts per ant, finding a suitable statistical test for these tendencies is difficult. One plausible test is a χ^2^ test against the hypothesis that the proportion of right-turning paths was the same before or after a left-turning and a right-turning saccadic bout; such a test would assume that decisions of each ant on each bout were independent. The inferential statistics then support what appears to be the case in Fig. S5, with significant effects for the path after but not the path before: (walk 1 before path, χ^2^=0.66, d.f.=1, *P*=0.41; walk 1 after path, χ^2^=21.1, d.f.=1, *P*<0.0001; walk 2 before path, χ^2^=0.53, d.f.=1, *P*=0.46; walk 2 after path, χ^2^=52.779, d.f.=1, *P*<0.0001). Stopping phases showed a minimum duration of ∼100 ms and a maximum up to 580 ms, with most of the distribution bunched at the short (left) end (Fig. 6). We also calculated the duration to complete the scanning bout of pirouettes and voltes (Fig. 7A). The sample size of voltes was small but voltes were on average longer than entire bouts of pirouettes.

From the videos, we also calculated the mean (±s.d.) time of scanning bouts from walk 1 and walk 2, measured from the start of the video recording, within 1 s of the appearance of the focal ant (Fig. 7B). In both walk 1 and walk 2, a linear fit accounts for most of the variance in average time of scanning bouts across bout numbers (*R*^2^_walk1_=0.98, *R*^2^_walk2_=0.99), with both intercepts slightly less than 0, meaning that bouts of scanning took place on average after a constant period of time. The slope was higher for walk 2, indicating a longer average interbout interval. Then, we calculated the distribution of durations of interbout intervals of walk 1 and walk 2 scanning bouts (Fig. 7C,D). The results show wide variability, including variability across bouts within individual ants. The Pearson correlation between scan duration and distance from nest of the scan location of walk 1 and walk 2 showed independence or a lack of correlation (*R*^2^_walk1_=−0.01, *R*^2^_walk2_=0.01) between scan locations and durations (Fig. S6A,B). The average duration of scans taking place in the four quadrants of walk 1 and walk 2 (Fig. S6C) was similar in each quadrant, with a repeated-measures ANOVA across the four quadrants showing no significance (*F*_3,62_=2.004, *P*=0.123).

**Fig. 8.**
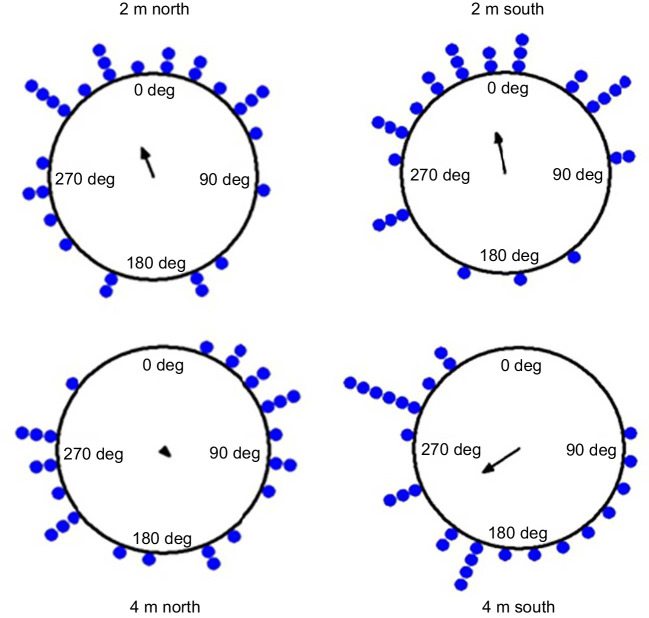
**Headings on displacement tests.** Circular histograms of initial headings of ants in the 2 and 4 m north and south displacement tests. The nest direction is set at 0 deg. Arrows denote the length and direction of the mean vector.

On a separate sample, ants trying to enter the nest after their naive (first) walk were captured and released on the goniometer at two different locations to the south and north of their nest entrance. The ants’ initial headings at each location showed that the majority of the ants in the 2 m displacements were oriented towards the nest direction of 0 deg. In contrast, ants that were displaced 4 m from the nest were not oriented towards the nest direction from the release point (Fig. 8). By the Rayleigh test, the foragers’ initial orientations were non-uniformly distributed in 2 m displacement tests whereas in 4 m tests they were scattered and uniformly distributed (Table 1, Fig. 8). In addition, ants in 2 m displacement conditions showed significant *V*-test results in the nest direction, and the means of their 95% confidence interval of initial heading values include the nest direction 0 deg (Watson test, *P*>0.05). The log likelihood ratio test for three tests (2 m north, 2 m south, 4 m north) failed to reject the hypothesis that the distribution was clustered in the home direction (*P*≥0.05, *k*≥0, χ^2^≤1). However, the 4 m south test rejected the hypothesis that the mean value of distribution was equal to the predicted value (home direction) (*P*=0.003, *k*=0.46 and χ^2^=6.6), meaning that the headings were oriented in a different direction from the nest direction.

**Table 1.**
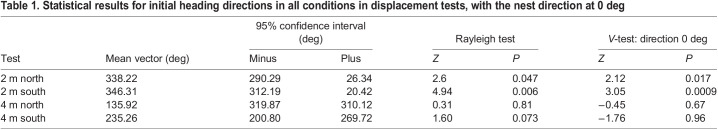
Statistical results for initial heading directions in all conditions in displacement tests, with the nest direction at 0 deg

## DISCUSSION

In summary, our observations of learning walks in *M. bagoti* showed that before becoming a forager, ants made 3–7 learning walks. The first walk of naive ants was close to the nest, short in duration, and covered only a small area. Over successive walks, ants covered a greater area, travelled a greater distance from the nest, and increased the duration of their learning walks. Stereotypical body rotations with stopping phases, pirouettes, increased across the first three walks as a result of the longer travel distance and then their numbers decreased. The stopping phases gave the ants multiple opportunities to view the nest location and its surroundings in different areas around the nest, likely contributing to learning the landscape. After the first learning walk, ants displaced 2 m away could orient towards the nest direction while ants displaced 4 m away could not do so, suggesting that a catchment area between 2 and 4 m had been learned in one walk in our setting. Our findings showed many parallels to learning walks in *Cataglyphis* ants (Fleischmann et al., 2016) and *Ocymyrmex* ants (Müller and Wehner, 2010), but also furnished additional walk-by-walk details.

Learning walks are characterised by the exploration of the area around the nest without getting any food. Studies on *Cataglyphis* desert ants and *M. croslandi* bull ants have revealed that they conduct a series of ever-increasing arcs around their nest in learning walks (Fleischmann et al., 2016; Jayatilaka et al., 2018). After 3–7 learning walks, *Cataglyphis* desert ants began to forage (Fleischmann et al., 2018a, 2016, 2018b; Wehner et al., 2004), whereas *M. croslandi* ants started foraging after 2–7 learning walks (Jayatilaka et al., 2018), similar for both species over the course of several days. *Melophorus bagoti* ants performed 3–7 learning walks over several days before foraging. Across the learning walks, individual ants tended to explore different compass directions around the nest and learning is expected to take place during this period. Thus, the number of learning walks before an ant goes foraging is similar across these diurnal species but differs between individuals within each species. It would be interesting to investigate what determines these individual differences in any of these species.

It is well known that desert ants form idiosyncratic routes leading them from their nest to foraging sites and back again, and these routes are unique to individuals and maintained over time (Kohler and Wehner, 2005; Mangan and Webb, 2012; Wystrach et al., 2020). Desert ants also show sector fidelity, foraging in a similar direction from one trip to another (Wehner et al., 2004). In our study, the centroid direction of the last learning walk of individual ants predicted the direction of an ant's first foraging trip (Fig. 2; Fig. S2). This suggests that sector fidelity develops even before the first foraging trip. We suppose that individual learners learned the views from particular directions especially well rather than focusing on panoramas from all directions.

Learning walks in the desert ant *C. fortis* have been described as three different types: slow choreographic walks that are ≤0.3 m in radius from the nest, short learning walks in the range 0.3–0.7 m and twisted exploratory walks ≥0.7 m in range (Fleischmann et al., 2016). In *C. fortis*, successive learning walks extend further from the nest, reaching a distance of up to 4 m from the nest. Our trial-by-trial observations showed that for *M. bagoti*, the first walks started slowly from the nest, attaining a ∼20 cm diameter loop in less than a minute, which is similar to the slow choreographic walks of *C. fortis* (Fleischmann et al., 2016). The second learning walks of *M. bagoti* mostly fit the middle category described by Fleischmann et al. (2016), while later walks fit the description of twisted exploratory walks (see Fig. 3; Fig. S1). Across successive learning walks, *M. bagoti* increase their area of exploration, maximum distance from the nest and walk duration, again much like *C. fortis* (Fleischmann et al., 2016).

Learning walks and learning flights in hymenopterans share common features where the animals rotate around their nest entrance before setting off to forage (honeybees: Becker, 1958; Capaldi and Dyer, 1999; Capaldi et al., 2000; Lehrer, 1991, 1993; Vollbehr, 1975; bumblebees: Hempel de Ibarra et al., 2009; wasps: Stürzl et al., 2016; Zeil, 1993a,b; wood ants: Judd and Collett, 1998; Nicholson et al., 1999; desert ants: Fleischmann et al., 2016; Müller and Wehner, 2010; current study). Over multiple flights or walks around their nest, wasps, bees and ants perform exploratory flights or walks, increasing in distance and widening their range of directions across trips (Capaldi et al., 2000). They explore all quadrants around the hive or nest across the exploration flights or walks, and the area that they cover around the nest increases progressively over successive learning flights and walks. In some rare instances, however, some bumblebees and honeybees explored all quadrants around the hive during a single flight (Capaldi and Dyer, 1999; Hempel de Ibarra et al., 2009). Bees show no substantial positive association between flight number and flight duration; as the bees gained experience, they tended to fly further by flying faster rather than longer (Capaldi et al., 2000). Here, the observations on *M. bagoti* desert ants showed that they, like other hymenopterans, also explore all the quadrants around the nest across all the learning walks. When flying wasps first leave their nest, they survey their nest in small arcs, backing away in sweeps while facing the nest (Zeil, 1993a, 1993b). Naive walks of *M. bagoti* desert ants also showed similarities with the learning flights of wasps, honeybees and bumblebees and the learning walk of *Cataglyphis* ants. The *M. bagoti* learner ants perform naive walks close to the nest, which last less than a minute, in a series of loops. While we lacked the equipment to quantify scan directions exactly, many scans were towards the general direction of their nest. Increasing the distance from home across trips and occasionally looking towards home are themes found in all these learning walks and flights.

In several ant species the characteristics of stereotypical stops and turn-back looks during learning walks have been reported. *Cataglyphis* ant species perform pirouettes and voltes. The stopping phases of pirouettes most likely serve in learning the distant panorama around the nest entrance, while the voltes are believed to provide the rotational movement that allows the celestial cues to be calibrated (Fleischmann et al., 2017). Our study revealed that *M. bagoti*, like *C. noda* and *C. aenescens*, performs both pirouettes and voltes. *Cataglyphis* ants turn their bodies to look back at the nest entrance during their walks (Fleischmann et al., 2017). The duration of scans in pirouettes is comparable across these species. As with *Cataglyphis*, the pirouettes likely also provide learning opportunities for *M. bagoti*.

We examined the temporal distribution of bouts of scanning and found that, on average, they occur after a fixed interval, a longer average interbout interval for walk 2 than for walk 1. What could generate this regularity? One possibility is a random generation process that produces each scanning bout at a random time but at a particular average rate, known as a Poisson process. In a Poisson process, the probability of a focal event taking place is constant at every moment in time. Bacteria (*Escherichia coli*) show a Poisson process in their tumbles or twiddles (Berg and Brown, 1972), which, like scanning bouts, interrupt normal movement. In bacteria, tumbles orient the organism to move in a (random) different direction (Berg and Brown, 1972). A Poisson process should generate an exponential distribution of interbout intervals, which is found in *E. coli*. The nematode *Caenorhabditis elegans* generates its version of tumbles, called simply turns, from two Poisson processes, producing a distribution of inter-turn intervals that can be described as the sum of two exponential functions (Srivastava et al., 2009). While the function describing interbout intervals in scanning bouts in *M. bagoti* remains uncertain, our preliminary data (see Fig. 7) look nothing like an exponential function, which should be bunched at the left (short) end. Another possibility is an oscillatory process. Such a process would produce a peaked distribution of interbout intervals with tails on either side, or possibly a distribution with multiple peaks if multiple oscillators operating at different frequencies are at play. Our preliminary data in Fig. 7 show wide variability within individual ants, making this hypothesis less likely, although the sample size was too small to rule out or support such hypotheses. A much larger corpus is needed to ascertain the nature of the distribution of interbout intervals. Such data could come not only from future studies but also from previously collected data on scanning bouts of *M. bagoti* and *Cataglyphis* ants.

We also plotted the distribution of stopping phases within scanning bouts (Fig. 6). While the sample size is again small, too small for confident curve fitting, in this case the distribution is bunched at the left end, resembling an exponential distribution. An exponential distribution would suggest a Poisson process that ends a stopping phase in pirouettes to move the ant to the next behaviour, either a rotation or walking again. The scan or pause duration did not depend on where the scan took place (Fig. S8), a finding suggesting an independent, possibly Poisson process governing scan durations. We urge scientists studying learning walks to report such distributional data because they address important theoretical questions. It would also be interesting to study in the future whether the kind of regularity in these behaviours depends on the environment around the nest.

Hymenopterans learn from their learning walks and learning flights. The ground-breaking findings of Becker (1958) and later experiments show that honeybees are able to find their home from places up to 200 m from their nest after a single orientation flight (Becker, 1958; Capaldi and Dyer, 1999). Three studies, on *M. bagoti* and *C. noda*, kept ants in a restricted area around the nest entrance for 2–3 days to test whether they generalise their views up to 10 m away at a site that they had never visited (Deeti et al., 2020; Fleischmann et al., 2018a; Wystrach et al., 2012). These experiments show that the ants had acquired sufficient information from their spatially restricted experience to home from the displacement sites. In the current study, on the particular nest located in an open area with a rich visual panorama (something that is also true of the displacement studies just cited), ants generalised to views 2 m from the nest after their first learning walk, although not to views 4 m from the nest. A more fine-grained trial-by-trial analysis of the learning process would be worthwhile as well as studies that compared the effects of the complexity and spatial frequencies of the visual environment.

To conclude, *M. bagoti* ants performed a sequence of distinctly choreographed learning walks in the vicinity of the nest before becoming foragers. These walks provide navigating ants with information about the surrounding panorama. As with *Cataglyphis* ants, the search range and duration of learning walks in *M. bagoti* increased across successive trips. Also paralleling *Cataglyphis* ants, *M. bagoti* exhibited pirouettes and voltes during their learning walks. From one learning walk, *M. bagoti* generalised the views that they learned to a distance of 2 m from their nest. Future studies should examine in more detail differences and similarities between species of ants in learning walks. The underlying neurological processes supporting learning walks also need to be examined.

## Supplementary Material

Supplementary information
