# Supplementary material for: Learning walks in an Australian desert ant, Melophorus bagoti
Source: J Exp Biol. 2021 Aug 26;224(16):jeb242177. doi: 10.1242/jeb.242177 (PMC8407660; doi:10.1242/jeb.242177)
Supplement: Supplementary information [file jexbio-224-242177-s1.pdf]

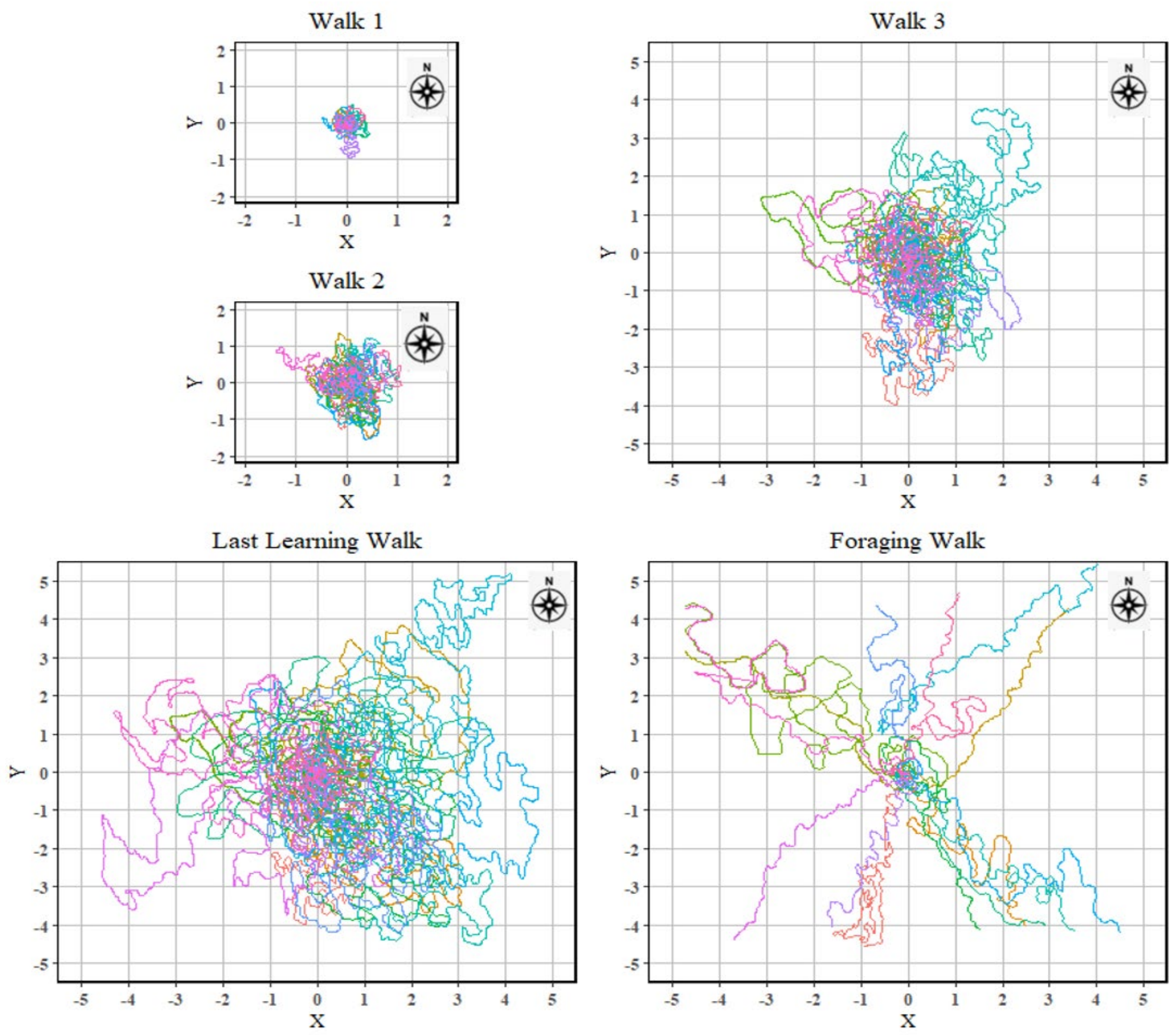

Fig. S1. **Successive learning walks of *Melophorus bagoti*.** Paths from Walk 1 to Walk 3 and the last learning walks and first foraging walks of all focal ants are shown on a 10 × 10 m grid. 0, 0 is the nest position. Each unit on the axes is 1 m. Each colour represents one ant.

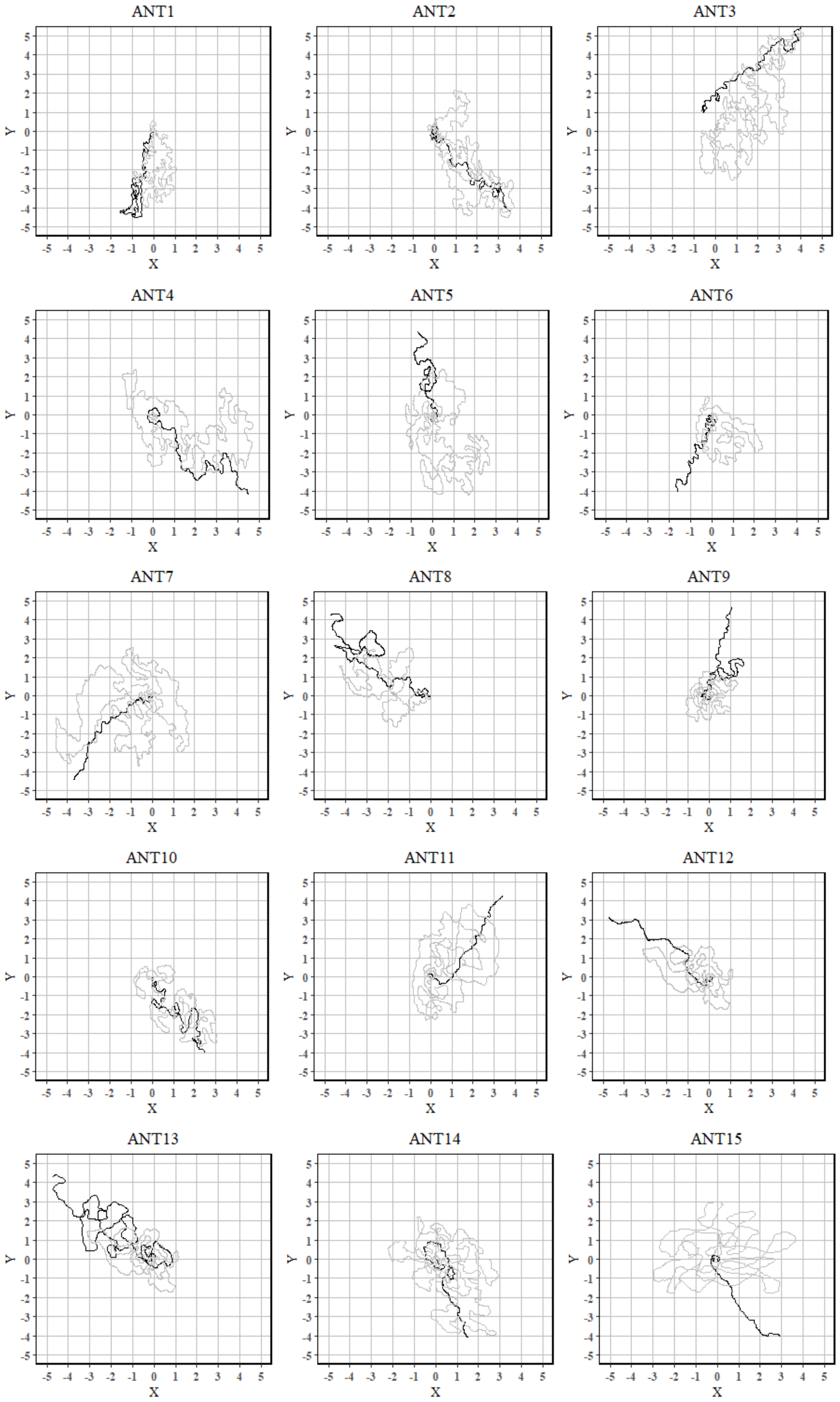
ANT1
ANT2
ANT3
ANT4
ANT5
ANT6
ANT7
ANT8
ANT9
ANT10
ANT11
ANT12
ANT13
ANT14
ANT15
X
Y
-5 -4 -3 -2 -1 0 1 2 3 4 5

Fig. S2. The last learning walks and first foraging walks. The last learning walks (grey) and foraging walks (black) of all focal ants are shown on a 10 × 10 m grid. 0, 0 is the nest position. Each unit on the axes is 1 m. Ant3's foraging path is not shown to start at 0, 0 because another ant was also performing a learning walking at the same time, so that the first portion of Ant3's foraging walk was not recorded.

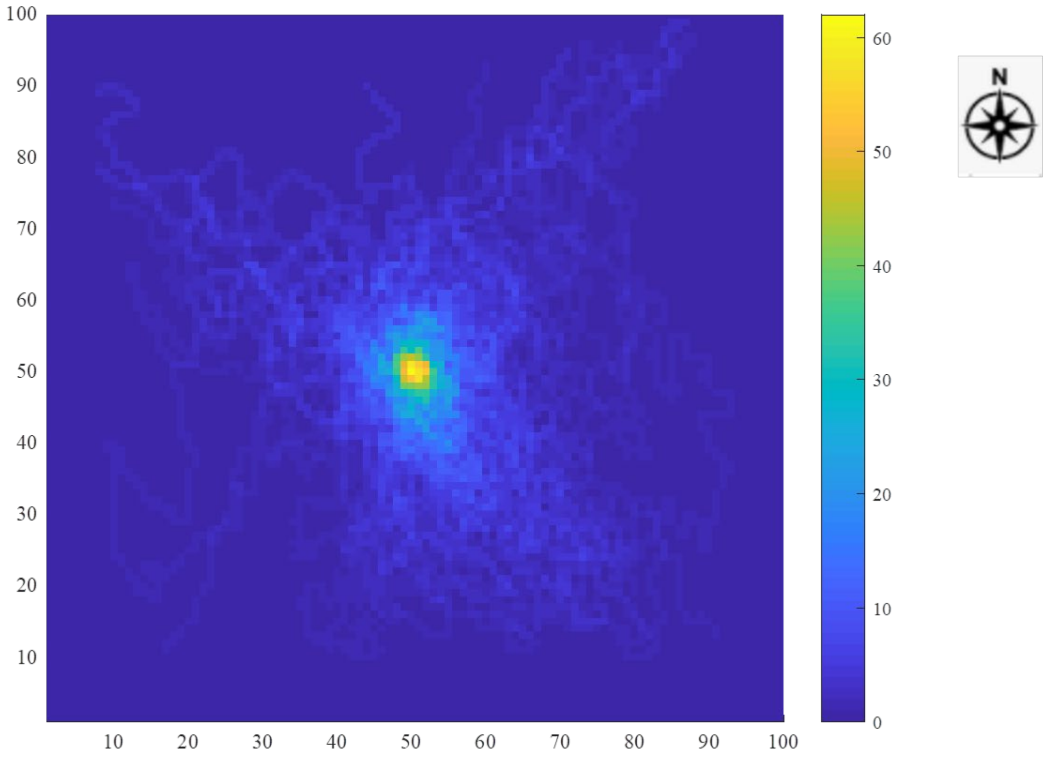

Fig. S3. Heat map of all learning walks. Heat-map showing the focal ants' travelled paths around the nest, pooling all walks of all ants. *X*- and *Y*-axis scale units of the false-colour map represent decimetres, encompassing the 10m × 10m gridded area around the nest at 50, 50. Dark blue pixels have not been visited by any ant, whereas yellow indicates many visits by ants. Next to the colour bar, the numbers represent the number of visits by ants across all ants and all walks, including repeat visits.

Journal of Experimental Biology • Supplementary information

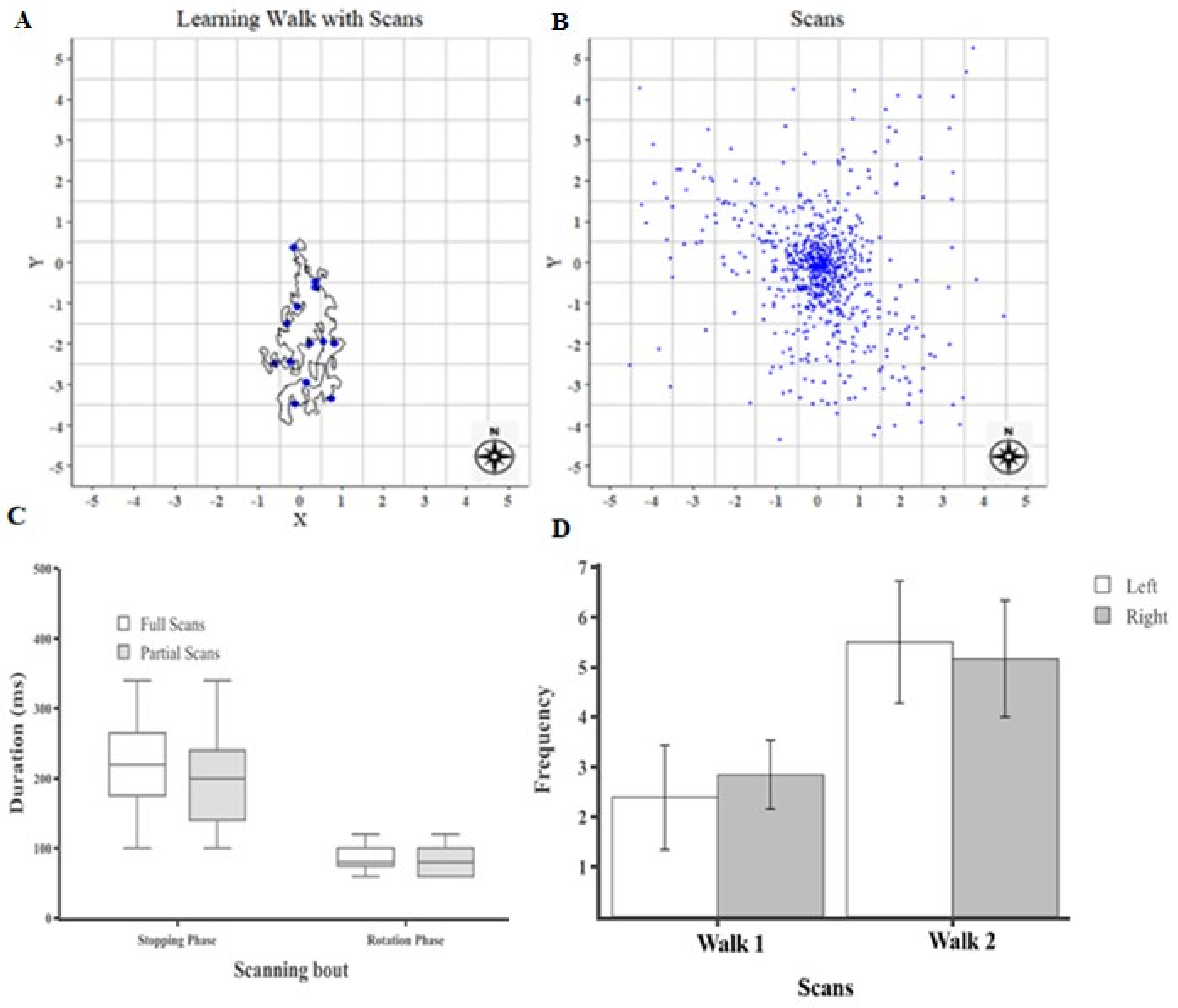

Fig. S4. Scan distribution, duration of scanning bouts and left and right turns in scanning. One sample learning walk of one *Melophorus bagoti* ant (Ant2) around the nest (A) showing positions of scanning bouts as blue dots and the positions of all scans across all the learning walks (B). The nest is located at 0, 0. The durations of stopping and rotation phases in scanning bouts captured on video **(C).** The box plots show the median (middle line in the box), the lower and upper quartiles of the box, and 1.5 times the interquartile range (whiskers). The average number of left and right turns during scanning bouts in the first (n=8) and second learning walks (n=6) with standard deviation (D).

Journal of Experimental Biology • Supplementary information

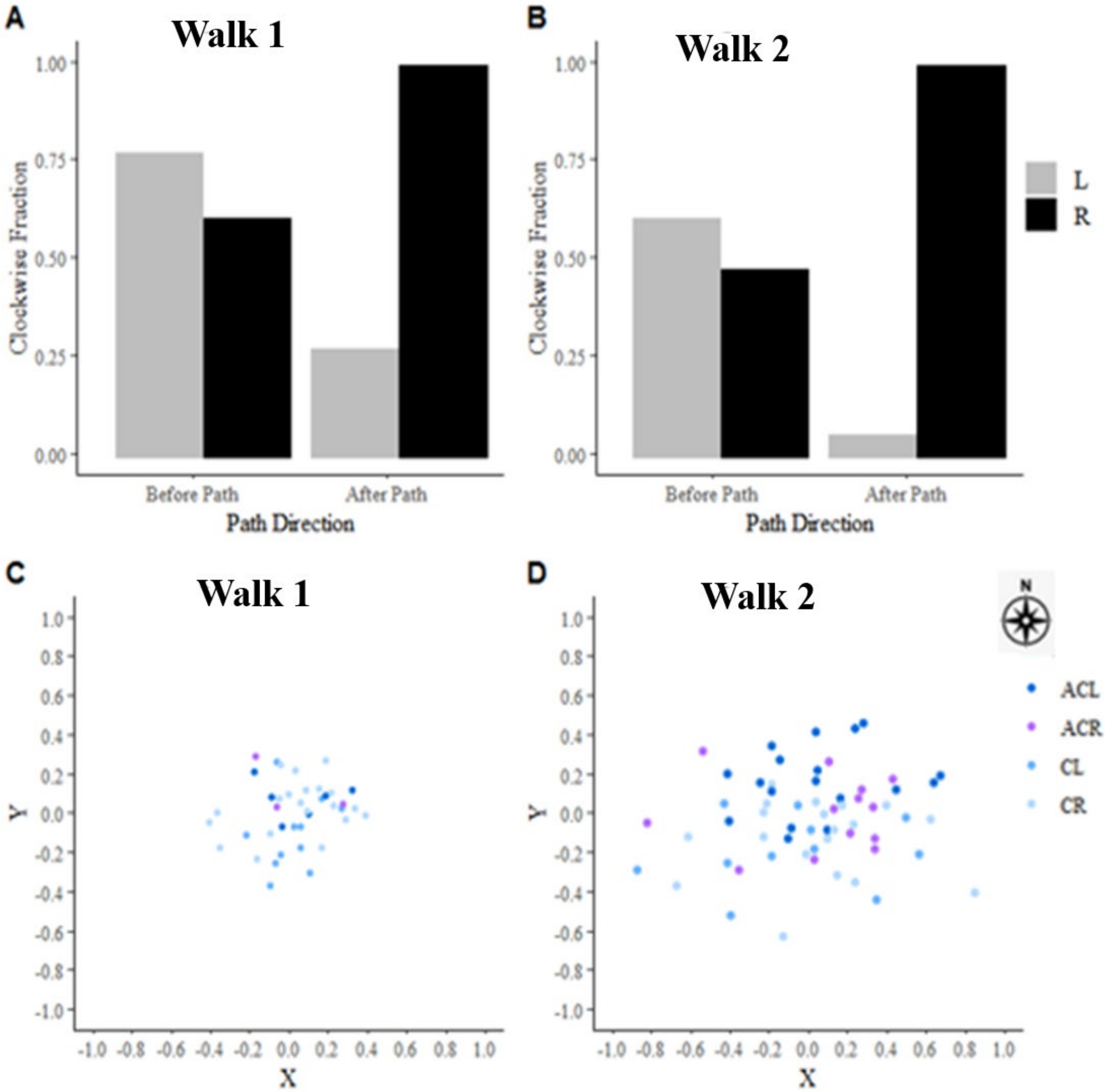

Fig. S5. The correlation between the curvature of paths and turning direction of scanning bouts. The turning direction of scanning bouts on Walk 1 and Walk 2 in relation to the curvature direction (to the left, L or to the right, R) of the path before and after the scanning bout (A–B). The *y*-axis shows the proportion of scanning bouts with saccades turning to the right (clockwise). The locations of clockwise and anticlockwise scanning bouts after left-turning or right-turning path segments leading up to the scanning bout on Walk 1 (C) and Walk 2 (D). ACL and CL represent anticlockwise and clockwise scanning bouts after a left-curving segment and ACR and CR represent anticlockwise and clockwise scanning bouts after a right-curving segment.

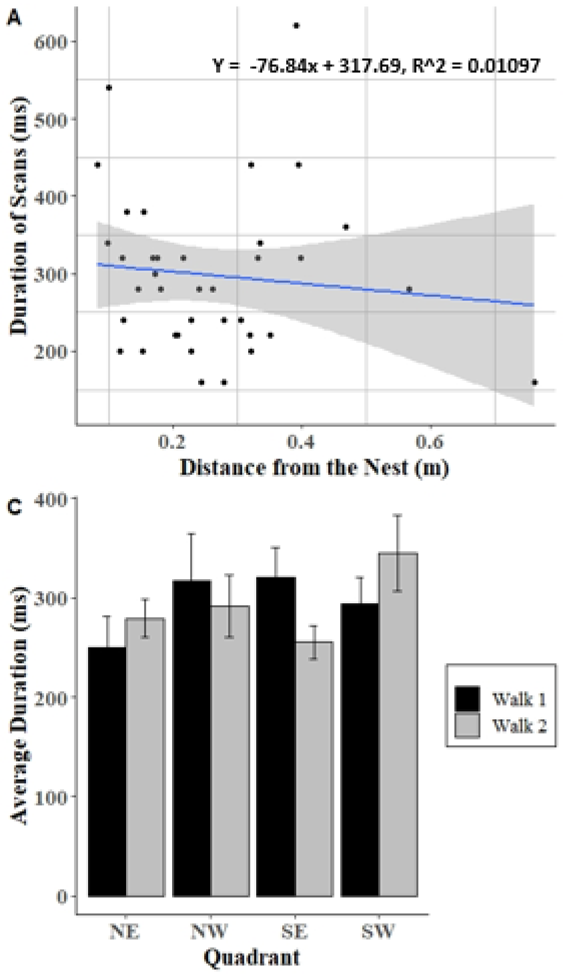

Fig. S6. Correlation between distance and duration of scans. The relationship between the distance from nest of a location of scanning and the scanning duration on Walk 1 (A) and Walk 2 (B). The average durations and standard error of scans taking place in the four quadrants of Walk 1 and Walk 2 (C). The reperated-measures ANOVA across the four quadrants showed no significance ($F_{3,62}$ =2.004, P=0.123). Each quadrant represents a 90° sector around the nest, Northeast (NE), Northwest (NW), Southeast (SE), and Southwest (SW).

Journal of Experimental Biology • Supplementary information
